# Immunophenotyping Reveals No Significant Perturbation to PBMC Subsets When Co-cultured With Colorectal Adenocarcinoma Caco-2 Cells Exposed to X-Rays

**DOI:** 10.3389/fimmu.2020.01077

**Published:** 2020-06-02

**Authors:** Giuseppina Borsci, Sofia Barbieri, Isabella Guardamagna, Leonardo Lonati, Andrea Ottolenghi, Giovanni Battista Ivaldi, Marco Liotta, Paola Tabarelli de Fatis, Giorgio Baiocco, Monica Savio

**Affiliations:** ^1^Laboratory of Radiation Biophysics and Radiobiology, Department of Physics, University of Pavia, Pavia, Italy; ^2^Unit of Radiation Oncology, ICS Maugeri, IRCCS, Pavia, Italy; ^3^Unit of Medical Physics, ICS Maugeri, IRCCS, Pavia, Italy; ^4^Immunology and General Pathology Unit, Department of Molecular Medicine, University of Pavia, Pavia, Italy

**Keywords:** Caco-2 cells, peripheral blood mononuclear cells, immunophenotyping, ionizing radiation, co-culture

## Abstract

*In vitro* co-culture models between tumor cells and peripheral blood mononuclear cells (PBMCs) allow studying the interplay between these cell populations, potentially gaining insight into the *in vivo* response of the immune system to the presence of the tumor, as well as to possible other agents as radiation used for therapeutic purposes. However, great care is needed in the experimental optimization of models and choice of conditions, as some setups might offer a limited possibility to capture subtle immune perturbations. A co-culture model of PBMCs from healthy donors and colorectal adenocarcinoma Caco-2 cells was successfully adopted in a previous work to measure effects on Caco-2 and modulation of signaling when these latter are irradiated. We here tested if the same experimental setting allows to measure perturbations to the main PBMC subsets: we performed immunophenotyping by means of flow cytometry and quantified helper and cytotoxic T cells, NK cells, and B cells, when PBMCs are cultured alone (control), in presence of non-irradiated Caco-2 cells or when these latter are exposed to a 10 Gy X-ray dose from a conventional radiotherapy accelerator. To measure a baseline response in all experimental conditions, PBMCs were not further stimulated, but only followed in their time-evolution up to 72 h post-irradiation of Caco-2 and assembly of the co-culture. In this time interval PBMCs maintain a high viability (measured via the MTT assay). Caco-2 viability (MTT) is slightly affected by the presence of PBMCs and by the high radiation dose, confirming their radioresistance. Immunophenotyping results indicate a large inter-individual variability for different population subsets already at the control level. We analyzed relative population changes and we detected only a small but significant perturbation to cytotoxic T cells. We conclude that this model, as it is, is not adequate for the measurements of subtler immune perturbations (if any, not washed-out by inter-individual differences). For this purpose, the model needs to be modified and further optimized e.g., including a pre-treatment strategy for PBMCs. We also performed a pooled analysis of all experimental observations with principal component analysis, suggesting the potential of this tool to identify subpopulations of similarly-responding donors.

## Introduction

Large efforts are being devoted to the study of the complex relationship between immune cells and developing tumors (1). The immune system can detect shifts from homeostasis when normal cells undergo malignant transformation and eliminate them (immunosurveillance, which is at the basis of immunotherapy applications), but neoplastic cells have the potential to proliferate despite the host response (immune evasion, a hallmark of cancer). Cancers can also inhibit the immune response (immunosuppression) or even pro-actively influence it to foster growth and invasion (immunosubversion). Interactions in the tumor microenvironment and stimulation of different lymphocytic subsets *in vivo* are at the basis of the final outcome (2, 3). It is also known that discrimination of different lymphocytic subsets can provide information that can be used as a variable with prognostic value, as it is the case for the intratumoral infiltration of natural killer (NK) cells in patients with different types of solid tumors (4) and e.g. (5, 6). *In vitro* experimental models as co-culture setups between tumor cells and peripheral blood mononuclear cells (PBMCs), including immunophenotyping of the lymphocytic pool, are useful tools to characterize underlying mechanisms. However, great care is needed in the choice and optimization of experimental models and choice of investigated conditions, as some setups might offer a limited possibility to capture subtle immune perturbations.

Recently released data from the International Agency for Research on Cancer (IARC) (7) reported on a global cancer burden that has risen to 18.1 million new cases and 9.6 million deaths in 2018, with colorectum cancer being among the three top cancer types in terms of incidence (10.2%, third after cancers of the lung and female breast), ranked second in terms of mortality. Colorectal cancer is clinically managed with either surgery, chemotherapy or radiotherapy (8), while it is one of the tumors in which immunotherapy has been shown less effective (9). With the aim of developing effective therapeutic combination strategies, the study of molecular mechanisms for immunogenicity in colorectal cancer is needed, also including consideration of how these can be affected by the administration of other therapeutic agents as drugs or radiation.

Caco-2 cells, derived from human colon adenocarcinoma, are able to differentiate when cultured on a porous membrane, creating a functional polarized monolayer that can be used as a model of intestinal barrier. Co-culture models of Caco-2 cells with other cell lines have been proposed, including studies on their interactions with the immune system (10). These latter have been mainly focused on the response of Caco-2 to exogenous stimuli and how this is modified in presence of cells of the immune system (11, 12). In our previous work (13) we adopted a co-culture model of Caco-2 cells and PBMCs from healthy donors. We focused our attention on the permeability of Caco-2 monolayer, the expression of tight-junction proteins, and on the cytokine release in the medium, in presence/absence of PMBCs and for sham- or X-ray exposed Caco-2 cells.

In this work we adopted the same experimental model, addressing the question of whether or not it is optimized enough to measure possible effects to PBMCs due to the presence of Caco-2 cells, both non-irradiated and exposed to a 10 Gy dose from a radiotherapy accelerator. PBMCs were obtained from blood draws from healthy male donors and were not further stimulated: we therefore measured their baseline response in the investigated experimental conditions, testing their possible activation starting from a quiescent state. PBMCs were found to maintain a high viability (MTT assay) up to 72 h post-irradiation of Caco-2 and assembly of the co-culture. We performed immunophenotyping via flow-cytometry, finally obtaining that no significant perturbation to the subsets of helper and cytotoxic T cells, NK cells and B cells can be measured in all investigated conditions. Based on these negative results, the enrolment of healthy donors was stopped at a relatively low number (nine) of subjects. For selected donors only, we also performed preliminary zymography measurements and cytokine analysis in co-culture media, confirming that the experimental model allows to characterize the modulation of signaling between the two cell populations when Caco-2 cells are irradiated, but it is not optimized to capture possible subtle perturbations of lymphocytic subsets that might follow.

## Materials and Methods

### Cell Culture and Co-Culture Setup

Colorectal adenocarcinoma Caco-2 cells (purchased from the American Type Culture Collection, ATCC) were used for the experiments. Cells were grown in T_75_ flasks (GreinerBio-One, Germany) in complete medium (RPMI 1,640 supplemented with 1% L-glutamine 2 mM, 1% Penicillin 100 IU/ml—Streptomycin 100 μg/ml and 10% Fetal Bovine Serum). Caco-2 cells were cultured at 37°C, 5% CO_2_, until 80–90% of the confluence was reached (7th and 20th passage). At this point cells were re-suspended and counted by an automatic Scepter™ counter (Merck Millipore, United States) through a 60 μm sensor and 2 × 10^5^ Caco-2 cells were seeded on a porous (0.4 μm) transparent membrane insert (ThinCert culture insert, GreinerBio-One, Germany) employed for the co-culture model. A week later, Caco-2 cells were differentiated to polarized monolayers (hence, at complete confluence) on the membrane inserts. Membrane inserts (after sham- or 10 Gy X-ray exposure, see below) were then placed in a 6-well plate containing PBMCs, thus establishing the co-culture between Caco-2 cells (apical) and PBMCs (baso-lateral compartment). The transparent porous membrane of the inserts allowed the exchange of signaling molecules between the two compartments but prevented Caco-2 cells from a direct interaction with PBMCs.

**Figure 1:**
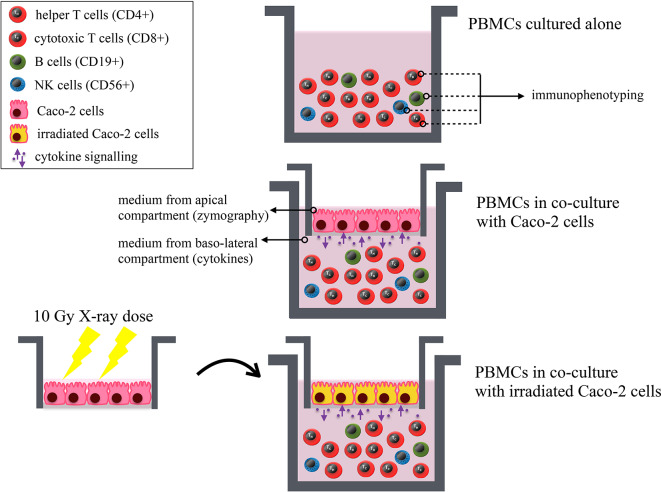
Schematic illustration of all investigated experimental conditions. Results on immunophenotyping of PBMCs from healthy donors are obtained for: PBMCs cultured alone; PBMCs in co-culture with Caco-2 cells; PBMCs in co-culture with Caco-2 cells irradiated with a 10-Gy dose of X-rays. The following lymphocytic subsets are identified: helper (CD4+) and cytotoxic (CD8+) T cells, B lymphocytes (CD19+) and NK cells (CD56+). Preliminary zymography measurements and cytokine analysis (Supplementary Material) are performed from the culture medium from the apical and baso-lateral compartment of the co-culture, respectively. The MTT assay is also performed on both cell populations in all investigated conditions. All endpoints are studied as a function of time.

Human PBMCs were obtained from heparinized peripheral blood through density gradient centrifugation using Ficoll (Histopaque-1,077, Sigma, USA). Blood draws from 9 healthy male donors were carried out at the Immuno-Hematology and Transfusion Medicine Department (S.I.M.T.), Diagnostic Medicine Department of *Policlinico San Matteo—Fondazione IRCCS*, Pavia, Italy. The study was carried out in accordance with the recommendations of the Ethical Committee of the *Policlinico San Matteo—Fondazione IRCCS*. The protocol was approved by the Committee. All subjects gave written informed consent in accordance with the Declaration of Helsinki. Cells were routinely cultured at 37°C in a humidified atmosphere containing 5% CO_2_. Human PBMCs were collected on the day of the experiment and 2 × 10^6^ cells/ml were put in the baso-lateral compartment of the co-culture 30 min after the irradiation of Caco-2 cells. All investigated experimental conditions are schematically illustrated in Figure 1.

### Irradiation Setup

The irradiation of Caco-2 cells was carried out at the Radiotherapy Department of ICS Maugeri (Pavia, Italy), using a 6 MV beam of a Varian Clinac 2,100™ (Palo Alto, USA) linear accelerator, routinely used to treat oncological patients. 6-well plates containing transparent 1 μm inserts (GreinerBio) with Caco-2 cells (for further assembly of the co-culture) were irradiated using a dose of 10 Gy, with a dose rate of 3 Gy/min at room temperature (RT). Control sham samples were prepared and underwent the same environmental/mechanical stresses as the other samples, except for the radiation exposure.

### Cell Viability—MTT Assay

The MTT [3- (4, 5-dimethylthiazol-2-yl)−2,5-diphenyltetrazolium bromide] (Sigma, USA) assay was performed to assess the viability of both Caco-2 and PBMC, when cultured alone or in co-culture with sham- or 10 Gy-irradiated Caco-2. The assay has been performed at 1, 24 and 72 h post-irradiation as previously reported (13). Data are expressed in terms of relative difference (%) in absorbance with respect to the 1 h time-point (and 0 Gy condition for Caco-2 cells), and then averaged.

### Immunophenotyping of PBMCs

For the analysis of PBMC subsets, an Attune™ NxT Acoustic Focusing Cytometer (ThermoFisher Scientific, US) available at the Radiation Biophysics and Radiobiology Laboratory (Physics Department, University of Pavia, Pavia, Italy) was used. Full details on the developed immunophenotyping panel used to characterize the main lymphocytic subsets are given in Supplementary Material, including antibodies/fluorophores with their dilution. Concentrations for the individual antibodies were chosen by means of titrations performed starting from the nominal values on the data sheets. Due to the limited PBMC extraction yield obtained with the Ficoll separation, the compensation matrix was implemented using control beads prepared with the AbC™ Total Antibody Compensation Bead Kit (Thermo Fisher Scientific, US). The flow-cytometry analysis was conducted using the Attune™ NxT Software. In Supplementary Material representative images of cytograms are shown, demonstrating the full gating strategy for the identification of lymphocytic subsets of interests. The first two gating steps concerns: (i) selection of the lymphocyte population from the FSC vs. SSC panel, related to the physical characteristic of the cells (size and granularity); (ii) singlet selection in the FSC-H vs. FSC-A panel, considering only events for which the FSC-H is proportional to FSC-A (diagonal of the panel). After these steps, the following lymphocytic subsets are identified in the population of CD45+ cells: helper (CD3+/CD4+) and cytotoxic (CD3+/CD8+) T cells, B lymphocytes (CD3–/CD19+) and NK cells (CD3–/CD56+). Application of a gate on CD45+/SSC was found to lead to compatible results. Data are finally given in terms of percentages of the subset population relative to the whole lymphocyte pool.

### Statistical Analysis

Experiments were repeated for the 9 different donors included in the study. For all experimental measurements involving PBMCs, it has to be considered that the number of experimental conditions investigated for a single donor was varied according to the limited yield of extracted PBMCs. For the same reason, technical replicates could not be foreseen. MTT results are averaged among donors (9 experiments), errors are presented as standard error of the mean (SEM). Immunophenotyping data are presented for individual donors. Relative changes in lymphocytic subsets are first calculated for each individual donor and then averaged, errors are presented as standard deviation. When shown, statistical significance (*p* < 0.05) was calculated by two-tailed Student's *t-*test.

### Principal Component Analysis

We considered as initial sets of variables for principal component analysis (PCA) all experimental observations for individual donors in terms of: lymphocytic subset percentages when PBMCs are cultured alone at different time-points; relative changes of all subset populations when PBMCs are co-cultured with unirradiated Caco-2 with respect to the control (PBMCs cultured alone) at a fixed time-point of 72 h. In the two cases we started, respectively, with a set of 12 and 4 variables. We then found the principal components, that are linear combinations of original variables, orthogonal to each other, thus simplifying the problem and avoiding redundant information (if any). As conventionally done, the first principal component is obtained as the variable maximizing the variance of the dataset, and each new principal component is chosen orthogonal to the previously defined one and adopting the same criterion. Each individual donor is represented by a point in the 2D space whose axes are the two principal components: PC1 and PC2 are defined from the initial set of 12 variables corresponding to four lymphocytic subsets at all three time-points; PC1' and PC2' are defined from the set of 4 ratios corresponding to relative changes in the four subsets at a fixed time-point. Transformation matrices to go from the initial set of variables to the chosen principal components are then applied to variables corresponding to the different experimental conditions: lymphocytic subset percentages when PBMCs are cultured with sham- and 10-Gy-irradiated Caco-2 in the first case; relative changes in subset populations when PBMCs are co-cultured with 10-Gy-irradiated Caco-2 with respect to the control (PBMCs cultured alone) at the same 72 h time-point. Donors in the different experimental conditions are represented as new points in the same principal component spaces, thus identifying possible clustering of donor groups or analyzing their repositioning in the mathematical space as an indicator of the response to the investigated condition. By construction, only individuals for which we have the same complete set of variables can be used in the PCA analysis. This leads to the consideration of 5 individuals only when analyzing absolute lymphocytic subset percentages. When considering relative changes of subset populations at the 72 h time-point, depending on the dose to Caco-2 cells, all 9 donors can be used for the analysis.

## Results

### Cell Viability

**Figure 2:**
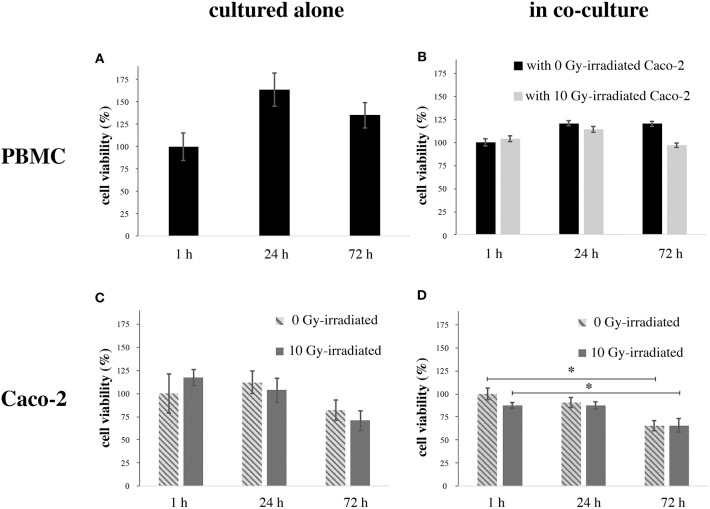
Viability (MTT) in PBMCs and Caco-2 cells as a function of time. **(A)** PBMCs cultured alone, normalized to the 1 h time-point; **(B)** PBMCs co-cultured with sham- or 10 Gy-irradiated Caco-2 cells, normalized to the 1 h time-point when in co-culture with non-irradiated Caco-2. **(C)** Caco-2, sham- or 10 Gy-irradiated, cultured alone, normalized to the 1 h time-point, 0 Gy; **(D)** Caco-2, sham- or 10 Gy-irradiated, in co-culture with PBMCs, normalized to the 1 h time-point, 0 Gy. Each value is the mean of 9 independent experiments, errors are expressed as SEM. Statistical significance (*p* < 0.05) is calculated by two-tailed Student's *t*-test.

Figure 2 reports cell viability data assessed with the MTT assay for both cell populations in all investigated conditions. Figure 2A reports data for PBMCs cultured alone, as percentages with respect to viability at 1 h. An increase in viability is observed after 24 h, followed by a decrease, with viability still higher than the control after 72 h. Figure 2B reports data for PBMCs co-cultured with Caco-2, when these latter are non-irradiated or irradiated at 10 Gy. Data are given as percentages with respect to PBMC viability at 1 h in presence of sham-irradiated Caco-2 cells. PBMC viability is slightly decreased below control level (to ~95%) only for the latest 72 h time-point and in case of co-culture with 10 Gy-irradiated Caco-2. Figure 2C reports data for Caco-2 alone, both sham- and 10 Gy-irradiated. Data are given as percentages with respect to Caco-2 viability in the 1 h, 0 Gy condition. A decrease in viability is observed only at 72 h, the effect being larger for 10 Gy- vs. non-irradiated cells (to ~70% vs. ~83%). Finally, Figure 2D reports data for Caco-2 (both sham- and 10 Gy- irradiated), when co-cultured with PBMCs. Viability is expressed relative to the 1 h, 0 Gy co-culture condition. Caco-2 viability tends to be lower with respect to cells cultured alone, in a time-dependent manner. In particular, in non-irradiated cells the viability decreases by 15 and 35% at 24 and 72 h, respectively; in irradiated cells, the viability is reduced at ~88% at 1 h, such value is not modified at 24 h and reaches the 65% 72 h post-irradiation.

### Immunophenotyping

**Figure 3:**
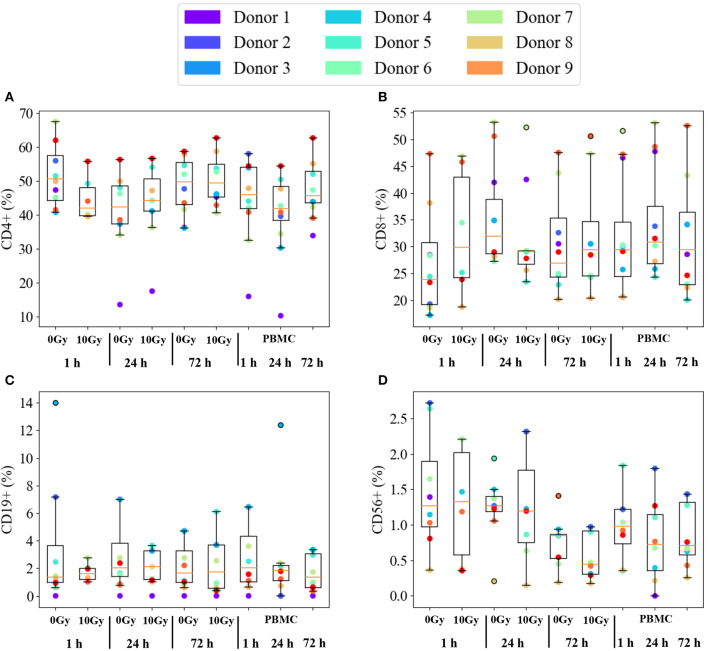
Immunophenotyping of PBMCs from individual donors. Subset population percentages for: **(A)** helper T cells (CD4+); **(B)** cytotoxic T cells (CD8+); **(C)** B lymphocytes (CD19+); **(D)** NK cells (CD56+). Experimental conditions are: PBMCs cultured alone or in co-culture with sham- or 10 Gy-irradiated Caco-2 cells, at the time-points: 1, 24, and 72 h. Colored dots are individual data. The median value is indicated by the red horizontal bar, the box is drawn from the first to the third quartile, with whiskers extending to the most extreme data points not considered outliers. Outliers (outside the +/−2.7 σ if data were normally distributed around the median) are plotted individually. Due to limited yields of extracted PBMCs not all conditions are present for each of the 9 donors.

Figure 3 reports immunophenotyping results for PBMCs from all 9 healthy donors in all investigated conditions. Data are expressed as percentage of each subset in the whole lymphocyte pool, subsets considered in the analysis are abbreviated as: helper (CD4+, Figure 3A) and cytotoxic (CD8+, Figure 3B) T cells, B lymphocytes (CD19+, Figure 3C) and NK cells (CD56+, Figure 3D). Individual data are reported as colored dots. For each condition, we also show the median and a box is drawn from the first to the third quartile. Dashed lines (whiskers) extend to the most extreme data points not considered outliers, and the outliers are plotted individually. Outliers are defined as those points outside the +/−2.7 σ (99.3% coverage) if data were normally distributed around the median value. The three conditions on the right-hand side of each panel refer to PBMCs cultured alone. Overall, no effect common to all donors can be observed on subsets as a function of time. The same holds when comparing the time evolution of PBMCs cultured with non-irradiated or irradiated Caco-2 cells (labelled, respectively, as 0 Gy and 10 Gy conditions). No clear pattern is evident also when looking at differences at the same time-point as a function of dose to Caco-2 cells.

**Figure 4:**
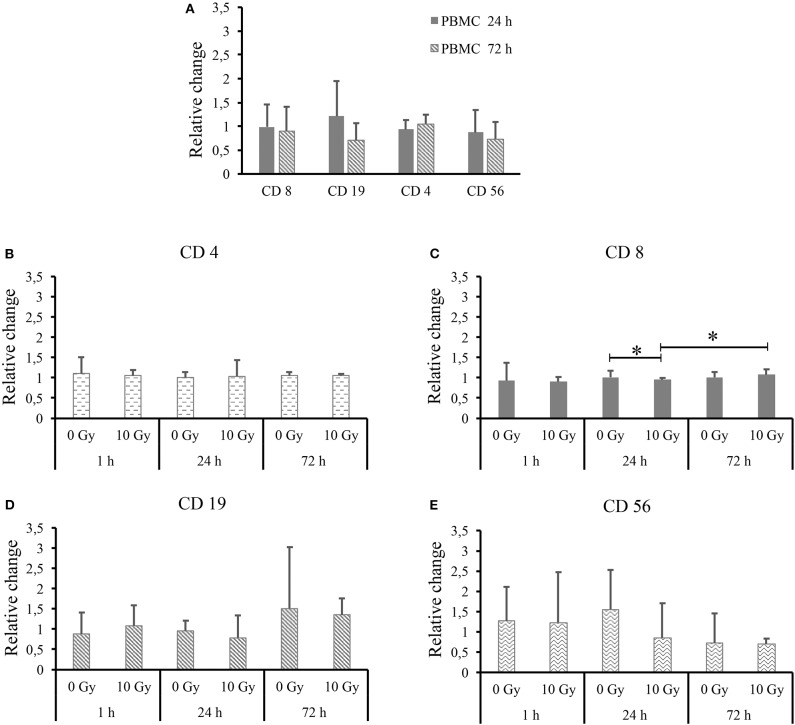
Average relative changes in PBMC subset populations for all donors. **(A)** For PBMCs cultured alone, relative changes in CD4+, CD8+, CD19+, and CD56+ subsets as a function of time with respect to the 1 h time-point. For PBMCs co-cultured with both non-irradiated and 10 Gy-irradiated Caco-2 cells, relative changes with respect to PBMCs cultured alone at the same time-point for subsets: **(B)** CD4+; **(C)** CD8+; **(D)** CD19+; and **(E)** CD56+. Ratios are calculated for individual donors (excluding outliers identified in Figure 3) and then averaged. Errors are obtained as standard deviations. Statistical significance (*p* < 0.05) is calculated by two-tailed Student's *t*-test.

Trying to highlight possible differences, the same dataset is further processed and shown in Figure 4. In particular, in Figure 4A we show all lymphocytic subsets for PBMCs cultured alone as a function of time, after normalization to the percentage measured for each subset at the 1 h time-point. Relative changes are calculated for each donor separately, thus allowing averaging over all donors. Figures 4B–E reports data for each lymphocytic subset (respectively, CD4+, CD8+, CD19+, and CD56+) when PBMCs are co-cultured with both non-irradiated and 10-Gy-irradiated Caco-2 cells, after normalization to the percentage measured for the same subset at the same time-point when PBMCs are cultured alone. Again, relative changes are calculated separately for each donor and then averaged. In all cases, outliers identified in Figure 3 are excluded from the analysis. For PBMCs cultured alone (Figure 4A) we do not measure relative changes in time of the percentage of different lymphocytic subsets, with the exception of a possible decrease in B lymphocytes, though ratios are affected by large variations. Throughout Figures 4B–E, most measured ratios are always consistent with a value of 1: overall, no significant perturbation of PBMC subsets is measured at equal time-points comparing PBMCs co-cultured with sham- and 10-Gy irradiated Caco-2 cells to PBMCs cultured alone. A small but statistically significant effect is observed for cytotoxic T cells only: at 24 h, with respect to PBMCs alone, no change in CD8+ lymphocytes is measured when PBMCs are co-cultured with sham-irradiated Caco-2 cells, while a slight decrease in the subset percentage is measured in the co-culture with 10 Gy-irradiated Caco-2 cells. Considering the time-evolution of the CD8+ subset when in co-culture with irradiated Caco-2 cells, an increase seems to be recovered at the latest 72 h time-point vs. the earlier 24 h one. Ratios for CD19+ and CD56+ lymphocytes are always affected by large statistical variations, and no conclusion can be drawn on the relative variation of such subsets as a function of time or co-culture conditions.

### Principal Component Analysis

**Figure 5:**
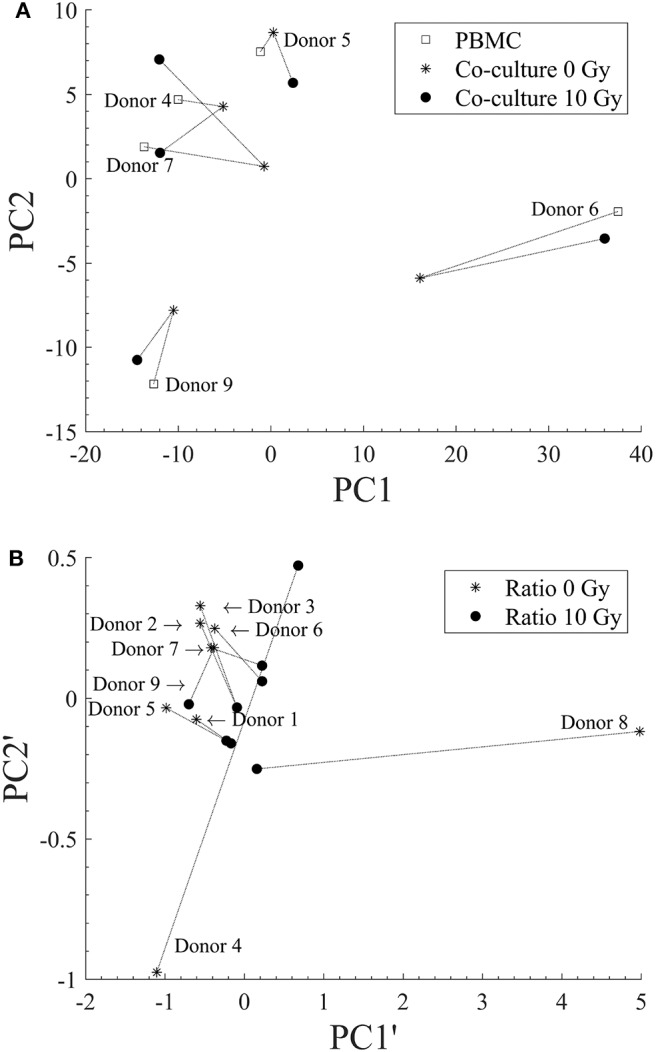
Principal Components Analysis for discrimination of donors for different experimental conditions. **(A)** PCA applied to lymphocytic subsets for each individual donor for: PBMCs cultured alone (squares); PBMCs in co-culture with sham-irradiated Caco-2 (stars); and PBMCs in co-culture with 10 Gy-irradiated Caco-2 cells (filled dots). **(B)** PCA applied to data at a fixed 72 h time point expressed as ratios between subset percentages of PBMCs when in co-culture with non-irradiated (star) or 10 Gy-irradiated Caco-2 cells (filled dots) and the same subset percentages when PBMCs are cultured alone. Dashed lines are drawn to guide the eye and connect points representing the same donors in different experimental conditions. Donors are identified by their numbers, the label is positioned close to the point representing PBMCs alone in **(A)** and the co-culture with non-irradiated Caco-2 cells in **(B)**.

In Figure 5A we show results obtained applying PCA to data on lymphocytic subsets for individual donors (original data in Figure 3) in the three experimental conditions: PBMCs cultured alone; PBMCs in co-culture with sham-irradiated Caco-2; and PBMCs in co-culture with 10-Gy-irradiated Caco-2 cells. In Figure 5B PCA is applied to data at a fixed 72 h time point, reprocessed and expressed as ratios between subset percentages of PBMCs when in co-culture with non-irradiated or 10-Gy-irradiated Caco-2 cells and the same subset percentages when PBMCs are cultured alone (original data in Figures 4B–E). In both panels, each individual condition for a single donor is represented by a point in the 2D space whose axes are the principal components (respectively, PC1, PC2 and PC1', and PC2'). Lines are drawn to guide the eye, connecting points representing the same donor in the different experimental conditions. In Figure 5A, no significant clustering of donors in the same experimental condition is observed and donors are always sparsely positioned in the principal component space, indicating a large inter-individual variability. A variability in the response can also be concluded observing the large repositioning in space when the experimental condition is changed for two out of five donors (Donor 6 and 7), while a much smaller repositioning is observed for the remaining ones. In Figure 5B, some degree of clustering can be observed for two subgroups of donors only (Donors 2, 3, 6, and 7 and Donors 1, 5), with a relatively small repositioning when Caco-2 are irradiated. The remaining two donors (Donor 4 and 8) are sparsely positioned in the space, and seem to be highly responding (large repositioning) to the presence of irradiated Caco-2 cells. This again suggests a high variability in the individual response.

## Discussion

Cell viability data assessed with the MTT assay for PBMCs indicate that the experimental culture conditions for fresh cells isolated from peripheral blood are good for the establishment of the co-culture up to the latest time-point (72 h). Considering that the MTT assay is a test for assessing not only cell toxicity and death but also cell proliferation, an indication about the capability of cell replication can be obtained. Other independent measurements performed on PBMCs from selected donors only confirm the cell viability data, and are found to be consistent with each other. In particular with the Trypan Blue assay [details on methods in (13, 14)] we obtain average percentages of dead PBMCs cultured alone of 1.8% and 10.7% at 24 and 72 h, respectively. When PBMCs are co-cultured with irradiated Caco-2 cells, a slight effect on their proliferation is observed only at 72 h. No significant impact on cell death is measured in this experimental condition, where the average percentage of dead PBMCs measured with the Trypan blue assay reaches its maximal value of about 10% at 72 h, comparable to PBMCs cultured alone at the same time-point.

Caco-2 cells are at confluence at the beginning of the experiment. Cells are not growing exponentially and no change in viability is expected because of growth. In the control condition (no irradiation and no co-culture) viability can decrease as a function of time only because of the lack of nutrients, as cells are not supplied with fresh medium during the course of the experiments. Considering the error bars, Caco-2 proliferation seems to overall decrease as a function of time (even if not appreciable at 24 h), both for sham- and 10-Gy-irradiated cells, and tends to be lower (with a decrease starting earlier) when cells are co-cultured with PBMCs. This can be partly attributed to the lack of nutrients and to cytokine signaling in presence of PBMCs. Overall, the high dose of radiation (10 Gy) seems to have a minor impact on cell proliferation, confirming their high radioresistance (13). Percentages of dead cells were previously measured with the Trypan Blue assay and found to increase in a dose-dependent manner, from ~10% for the sham to ~20% for 10 Gy-irradiated Caco-2 cells cultured alone at 48 h after exposure (13).

Inter-individual variations seem to dominate the immunophenotyping results in Figure 3. It is known that subset populations in peripheral blood of healthy individuals vary according to gender, age and ethnic groups. Several in-depth immunophenotyping campaigns have been conducted to recommend reference ranges for lymphocyte population parameters, which is needed to evaluate individual patients if further disease-related variations are to be consistently assessed. Donors selected for this study were all males over 18. Subset population data derived from PBMCs cultured alone are taken as reference to investigate possible further effects related to time, co-culture with and radiation dose to Caco-2. Individual percentages of helper and cytotoxic T cells, B lymphocytes and NK cells are found to vary in large ranges, but Figure 3 helps appreciating that this is partly due to characteristics of single donors that can be identified as outliers. Median values among all donors in control conditions compare relatively well to reference ranges measured for Caucasian male individuals of age below 50 (15) considering helper and cytotoxic T cells (respectively, the median value for CD4+ is found to be ~46%, in the range 27–52%, and the median value for CD8+ is ~29%, in the range 11–40%). Instead, measured percentages for B lymphocytes and NK cells are generally lower than reference values (the median value for CD19+ is ~2% and the range is 8–24%; the median for CD56+ is ~1% and the range is 4–27%).

Even if outliers are excluded from the analysis, subset populations vary in relatively large ranges, which suggests that averaging over different individuals is not a proper strategy to investigate possible effects on the clusters of differentiation in the investigated experimental conditions. Data were therefore reprocessed calculating relative variations in subset percentages for each individual donor first, before averaging (Figure 4): when PBMCs are cultured alone, no significant effect of time is observed, looking at the ratio between the subset population at each time-point and the same subset population at 1 h; when PBMCs are co-cultured with Caco-2, no clear effect of the presence of sham- or 10-Gy-irradiated Caco-2 is observed, looking at the ratio at equal time-point between the subset population in PBMCs in co-culture and the same subset population in PBMCs alone. Only cytotoxic T cells seem to be slightly affected by the presence of Caco-2 cells, their population being decreased when in co-culture with 10-Gy irradiated Caco-2 cells at 24 h, but recovering to increase at the latest 72 h time-point. The perturbation to cytotoxic T cells seems therefore strong enough not to be washed-out by inter-individual differences. It has to be stressed here that results presented in this work are obtained with a limited number of donors (nine), who were also not sorted based on their HLA typing. Generally speaking, it is acknowledged that a minimum of about 25 donors is required to cover most of the genetic variability of immune response (16). Also, the presence of single or multiple HLA matching/mismatching between the single donor and Caco2 cells, could differentially impact the stimulation of T and NK cells. However, though small, the changes we measured in the cytotoxic T cell subsets in the above-mentioned experimental conditions are found to have statistical significance. The limited number of donors and the differential background in PMBCs act as confounding factors (as in all studies of this kind) that might make other minor perturbations (not washed-out by inter-individual differences, if any) unmeasurable with this experimental setting. Enough data are therefore reported to conclude that our model seems not optimal to capture immune perturbations. As we later discuss though, this is more to be attributed to the choice of measuring the baseline response of PBMCs, without any prior stimulation, rather than to the statistical power of the study.

A pooled analysis of all experimental observations with principal component analysis also indicates that no clear common behavior can be observed among all donors (Figure 5), neither considering donor data in terms of absolute percentages of the different subsets, nor considering relative changes in subset populations. However, when data are analyzed in terms of relative changes, some degree of clustering of donors in the principal components space is observed, suggesting that this strategy is more suited to detect possible effects in spite of a large inter-individual variability. Interestingly, in the possible different principal component spaces that can be created, some donors seem to show a scarce sensitivity to the different experimental conditions, while others seem to respond with a net repositioning, but without a common pattern. This is also suggesting individual variability in the response to the different experimental conditions. Increasing the number of donors could both allow the identification of donor subpopulations and the study donor-related effects, and we show that PCA is a powerful tool to be exploited for this purpose.

In our previous work (13), using the same co-culture model, we showed that cytokine signaling from Caco-2 is differentially modulated depending on the presence of PBMCs and on radiation dose. In particular, considering the effects of measured interleukins, we could expect effects on immune cell proliferation (related to IL-2 signaling), effects on the deregulation of the Th1 and Th2 subsets of CD4+ cells (related to IL-4) and more specifically on the proliferation of NK cells (related to IL-15 signaling). Deregulation in Th1 and Th2 subsets of CD4+ T cells in peripheral blood of colorectal cancer patients has been highlighted in (17). Intratumoral infiltration of NK cells has also been measured in patients with colorectal cancer, and found to be associated with a favorable outcome in patients with TNM Stage III disease (5). Indeed, stimulation of NK cells (by upregulation of certain NK ligands) can lead to cancer eradication during tumorigenesis and metastasis (2, 18, 19). Though the setup here under study involves colorectal cancer cells with a poorly aggressive tumor phenotype and does not allow the study of lymphocyte infiltration, a change in the population of NK cells could be speculated as a consequence of signaling in the co-culture. Effects on PBMC viability could also be expected based on the measurement of a modulation of TNF-α signaling in co-culture, always in (13). Preliminary measurements to characterize the cross-talk between Caco-2 and PBMCs were also performed for this work: for two donors (selected randomly out of the 9) we measured cytokines in the culture medium from the baso-lateral compartment, and gelatinase B (MMP-9) production in the culture medium from the apical compartment in the co-culture setup with sham- or 10-Gy- irradiated Caco-2 cells, at 24 and 72 h post-irradiation and/or assembly of the co-culture. MMP-9 is synthesized from alveolar macrophages, polymorphonuclear leukocytes and osteoclasts (20). The expression of gelatinase B increases during the onset of inflammatory bowel diseases also in different experimental models, resulting in the most expressed protease in these pathological conditions (21). Results of these measurements are reported in Supplementary Material. Overall, published data (13) as well as these preliminary observations confirm the modulation of signaling in the co-culture when Caco-2 cells are irradiated, but in a way that also appears to be subject to inter-individual variations and does not induce significant changes in lymphocytic subsets that can be measured within the investigated time-frame using our experimental model.

Concluding, the main finding of this study is that the experimental model we adopted seems not optimized to capture immune perturbations when PBMCs are co-cultured with Caco-2 cells, also when these latter are exposed to a X-ray dose of 10 Gy. With the same model, in a previous work (13), we had focused on effects to Caco-2 and modulation of the signaling between cell populations in the same experimental conditions. We therefore wanted to measure data on effects to immune cells that could be eventually integrated with the already acquired knowledge on the same system. As a clue to understand the lack of measured effects, it is important to recall that most of the works in the literature addressing how lymphocytic subsets are perturbed following exposure to different agents (as well as to cancer cells) adopts protocols with pre-stimulation of PBMCs with polyclonal activators such as LPS, mitogenic lectins or antibodies [see e.g., (22–24)]. Differently, in this and our previous work, PBMCs were not stimulated, but only followed in their time-evolution up to 72 h, to measure their baseline response to the presence of Caco-2 and to the exposure of these latter to a high radiation dose: our results indicate that this is not enough for PBMC activation. However, a differential modulation of PBMCs—Caco-2 signaling is measured with the same model, which remains a good *in vitro* tool to characterize the cross-talk between tumor cells and cells of the immune system [see (13) and data in Supplementary Material for this work]. This suggests to perform additional studies with an alternative experimental design, as e.g., the use of a conditioned medium as PBMC activator (3) (without resorting to exogenous agents as LPS) or their exposure to a lower priming radiation dose (25, 26) (also mimicking exposure of circulating blood during radiation therapy), which could make the same model suited to study possible effects on the clusters of differentiation in the lymphocyte pool. An alternative strategy could also foresee the use of a similar *in vitro* experimental setting with myeloid cell subsets, also known to act as tumor sensor.

The large inter-individual variations dominating immunophenotyping results, and the inter-individual differences in the modulation of the signaling, also provide a clear indication to increase the number of healthy donors to be considered for future experiments. An alternative experimental setting including PBMC pre-treatment, the inclusion of more donors and the application of exploratory data analysis strategies for donor classification (as PCA, tested in this work) are to be considered for a follow-up of this work and the establishment of an *in vitro* co-culture model able to capture immune perturbation due to the presence of cancer cells and use of radiation as a therapeutic agent.

## Data Availability Statement

The datasets generated for this study are available on request to the corresponding author.

## Ethics Statement

The studies involving human participants were reviewed and approved by Ethical Committee of the Policlinico San Matteo—Fondazione IRCCS, Pavia, Italy. The patients/participants provided their written informed consent to participate in this study.

## Author Contributions

GBo, SB, AO, GBa, and MS: conceived the experiments. GBo, SB, IG, and LL: performed the experiments. GBo, SB, GI, ML, and PT: carried out sample irradiations at the Radiotherapy Department of the *IRCCS S. Maugeri* (Pavia, Italy). GBo, SB, IG, LL, GBa, and MS: performed data analysis and data interpretation. GBo, SB, GBa, and MS: wrote and edited the manuscript. AO, GBa, and MS: critically read the manuscript.

## Conflict of Interest

The authors declare that the research was conducted in the absence of any commercial or financial relationships that could be construed as a potential conflict of interest.
