# Supplementary material for: Immunophenotyping Reveals No Significant Perturbation to PBMC Subsets When Co-cultured With Colorectal Adenocarcinoma Caco-2 Cells Exposed to X-Rays
Source: Front Immunol. 2020 Jun 2;11:1077. doi: 10.3389/fimmu.2020.01077 (PMC7326036; doi:10.3389/fimmu.2020.01077)
Supplement: Supplementary file 1 [file Data_Sheet_1.pdf]

Supplementary Material

1. SUPPLEMENTARY INFORMATION ON METHODS

1.1 Immunophenotyping of PBMCs

For the analysis of PBMC subsets, an Attune™ NxT Acoustic Focusing Cytometer (ThermoFisher Scientific, US) available at the Radiation Biophysics and Radiobiology Laboratory (Physics Department, University of Pavia, Pavia, Italy) was used. **Table 1S** reports details on the immunophenotyping panel used to characterize the main lymphocytic subsets, including antibodies/fluorophores with their dilution. **Figure 1S** shows the full gating strategy, with representative images of cytograms. The first two gating steps concerns: i) selection of the lymphocyte population from the FSC vs. SSC panel, related to the physical characteristic of the cells (size and granularity) **(Figure 1S A)**; ii) singlet selection in the FSC-H vs. FSC-A panel, considering only events for which the FSC-H is proportional to FSC-A (diagonal of the panel) **(Figure 1S B)**. After these steps, the following lymphocytic subsets are identified in the population of CD45+ cells **(Figure 1S C)**: helper (CD3+ / CD4+) and cytotoxic (CD3+ / CD8+) T cells **(Figure 1S D)**, B lymphocytes (CD3- / CD19+) and NK cells (CD3- / CD56+) **(Figure 1S E)**. Application of a gate on CD45+ / SSC was found to lead to compatible results. Data are finally given in terms of percentages of the subset population relative to the whole lymphocyte pool.

Probe	Antibody	Fluorophore	Dilution	Channel
**CD45**	Monoclonal Antibody (HI30)	Pacific Orange (Life Technologies, USA)	1:40	VL3
**CD4**	Monoclonal Antibody (SK3 (SK-3))	Super Bright 702 (Invitrogen Corporation, USA)	1:40	VL4
**CD3**	Clone SK7	FITC (BD, Franklin Lakes, New Jersey, USA)	1:80	BL1
**CD56**	Monoclonal Antibody (MEM-188)	PE (Invitrogen Corporation, USA)	1:40	BL2
**CD8**	Monoclonal Antibody (3B5)	PE-Cyanine5.5 (Invitrogen Corporation, USA)	1:40	BL3
**CD19**	Monoclonal Antibody (SJ25-C1)	PE-Cyanine7 (Invitrogen Corporation, USA)	1:40	BL4

TABLE 1S | Details on the immunophenotyping panel.

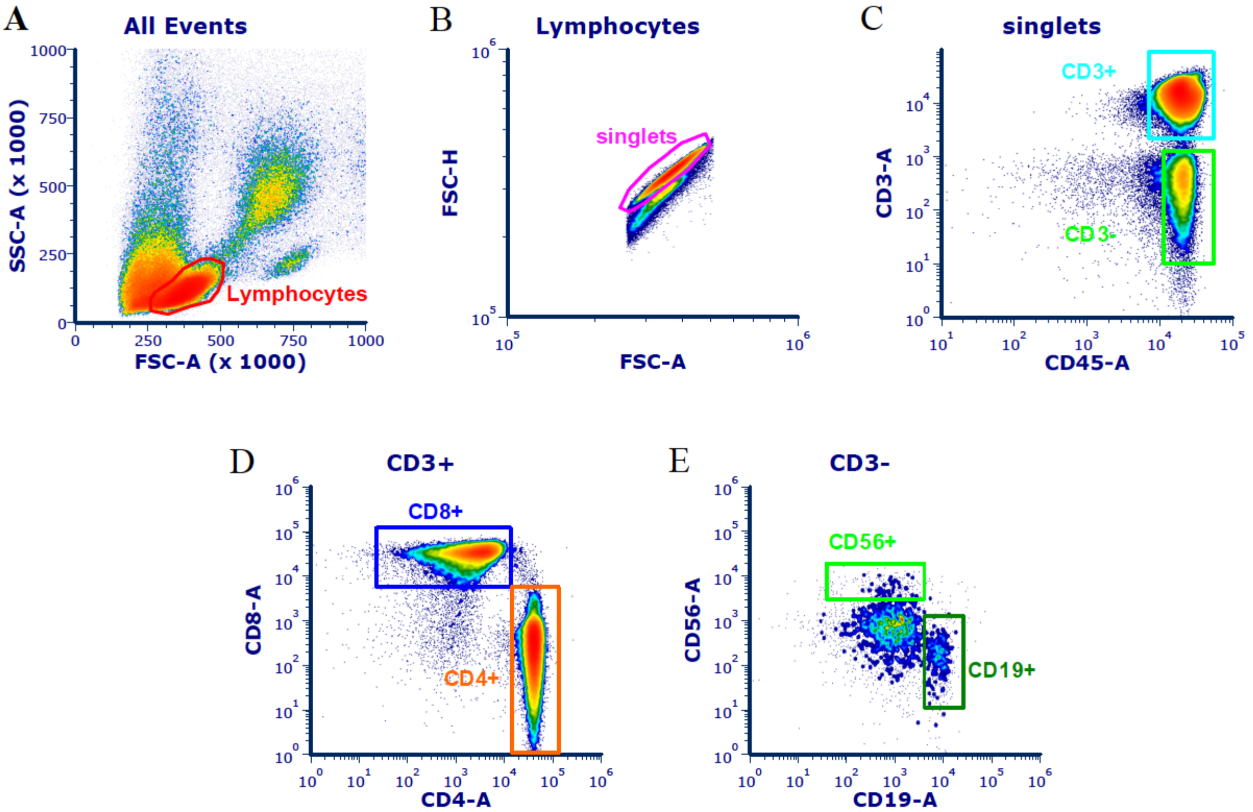

FIGURE 1S | Representative images of cytograms demonstrating the gating strategy for the identification of lymphocytic subsets. (A) selection of the lymphocyte population from the FSC vs. SSC panel; (B) singlet selection in the FSC-H vs. FSC-A panel; (C) discrimination of CD45+ / CD3+ and CD45+ / CD3- cells; (D) discrimination of helper (CD3+ / CD4+) and cytotoxic (CD3+ / CD8+) T cells; (E) discrimination of B lymphocytes (CD3- / CD19+) and NK cells (CD3- / CD56+). Obtained with *FCS Express* for illustration purposes.

1.2 Zymography

Preliminary measurements of Matrix Metalloproteases (MMP-9) in the culture medium from the apical compartment of the co-culture were performed following the experimental procedure already published in (1) with minor changes. The analysis was carried out for 2 donors (Donor 4 and Donor 5), selected randomly out of the 9 considered in the study. The conditioned media were collected after 1, 24 and 72 h of PBMC and sham- or 10-Gy-irradiated Caco-2 co-culture. Supernatants were stored at -20°C immediately after the disassemblement of the co-culture and the day before the analysis were centrifuged and mixed with equal amounts of sodium dodecyl sulphate (SDS) sample buffer. 30 µl of each sample was loaded on a 10% polyacrylamide gel containing 1 mg/ml Bovine Type B Gelatin (Sigma-Aldrich). Gels were stained with Coomassie Blue R-250 (0.5%w/v) and subsequently de-stained and acquired with Image Gel Analyzer (Bio-Rad).

1.3 Cytokine analysis

Preliminary measurements of cytokines in the culture medium from the baso-lateral compartment of the co-culture were carried out using the Human Cytokine Antibody Array (Abcam, Cambridge, UK),

according to the manufacturer instruction. The kit allows the simultaneous quantification of 23 different cytokines related to pro-inflammatory pathway. Culture medium was collected 24 and 72 h post-irradiation for PBMCs in co-culture with both sham- and 10 Gy-irradiated Caco-2 cells, for the same donors as in the metalloproteases assay. Images of treated membranes were acquired with the Azure c600 (Azure Biosystems, Inc, US) system, available at the Department of Molecular Medicine (University of Pavia, Pavia, Italy). Identification of regions of interest and intensity quantification were performed with the ImageJ plugin Protein Array Analyzer for ImageJ (Gilles Carpentier). Results are expressed as fold changes (FC) between the 10 Gy-irradiated and sham condition at the two different time-points.

2. SUPPLEMENTARY DATA AND DISCUSSION

Investigations of metalloproteases and cytokines were performed for two donors selected randomly out of the 9 included in this study (Donor 4 and Donor 5). The aim of these supplementary measurements was to confirm the validity of the co-culture model to measure the modulation of signaling between the apical (Caco-2) and the baso-lateral (PBMCs) compartments, when Caco-2 cells are exposed to radiation.

We focused on Matrix Metalloproteases MMP-9 **(Figure 2SA)**, that activates several signaling molecules and pathways during inflammation and cancer, while it is inactive in normal conditions (2). Gelatin zymography exploits the gelatinase activity of MMP-9, allowing to detect with high sensitivity the matrix degradation (white bands). Looking at **Figure 2SA**, a difference in the response of the two donors is clearly observed: the gelatinase activity of MMP-9 is demonstrated by the white band observed only in Donor 5 at 72 h, when PBMC are co-cultured with Caco-2 exposed to 10 Gy. Concerning cytokine secretion, we considered a panel of 23 cytokines and chemokines (**Figure 2SB**), related to pro-inflammatory pathway. We focused in the discussion on those showing potential correlation with MMP-9 activity, and those possibly related to immune cell perturbations. Cytokine results are shown as fold changes (FC) between the 10 Gy-irradiated and sham condition at the two different time-points, 24 h (blue) or 72 h (orange).

FCs have opposite signs for the two donors for both time-points for TGF-β and IFN-γ, and for the latest 72 h time-point for GRO-α. Concerning IL-1 α, a decrease in media from co-cultures with irradiated with respect to non-irradiated Caco-2 cells is observed for both donors, but comparing the two time-points the two donors behave differently. MMP-9 expression was found to be inversely correlated with IL-1α in corneal epithelium (3); despite the different cellular type, this is coherent with a negative fold change at the same time-point, indicating a decrease in IL-1 α for 10-Gy- vs. sham-irradiated Caco-2, though this is actually measured for both donors. Moreover, it is known that $CD8^+$ lymphocytes (Tc) produce IFN-γ and IL-2 (in turn, enhanced by IL-6 activity on lymphocytes) (4); we observed in both donors at 72 h an increased IL-2 signal, suggesting the activation of Tc lymphocytes, while an increased MMP-9 signal was also correlated with a decrease of TGF-β and of IFN-γ, supporting IFN-γ suppressing activity on MMP-9 mediated by JAK/STAT pathway (5). MMP-9 also acts on the degradation of chemokines such as GRO-α (6). These findings are coherent with negative fold changes for TGF-β, IFN-γ and GRO-α at the 72 h time-point, measured for Donor 5 but not for Donor 4. Concerning other lymphocytic subsets, IL-10 inhibits production of different inflammatory mediators (*e.g.* IFN-γ by T_H1 lymphocytes, IL-4 and IL-5 by T_H2 lymphocytes, IL-6 and IL-8 by mononuclear phagocytes and TNF-α by NK cells) (4). A different trend is found for the two donors for IL-10, which seems to be consistent with the lack of a common effect measured via immunophenotyping, due to the fundamental role of IL-10 in the regulation of many other cytokines produced by T_H and NK. Another cluster of chemokines included in the panel are monocyte chemoattractant proteins (MCP), responsible for the enhancement of IL-4; measuring levels of MCP-

1, MCP-2 and MCP-3, only for MCP-2 a similar trend for both donors was observed, but this is probably not enough to induce T_H2 phenotype.

Overall, measured effects indicates modulation of signaling between apical and baso-lateral compartment, and the production of MMP-9 by Caco-2 cells seems strictly correlated with inflammatory mediators released also by PBMCs. PBMCs themselves receive signaling molecules from X-ray- exposed Caco-2 cells, with a signal modulation that is necessarily time-dependent.
Though performed only for two donors, these measurements indicate that signaling modulation can be measured with this setup, but happens in a way that also appears to be subject to inter-individual variations and does not lead to measurable perturbations to PBMC subsets in this experimental setup, as concluded in the main work.

A

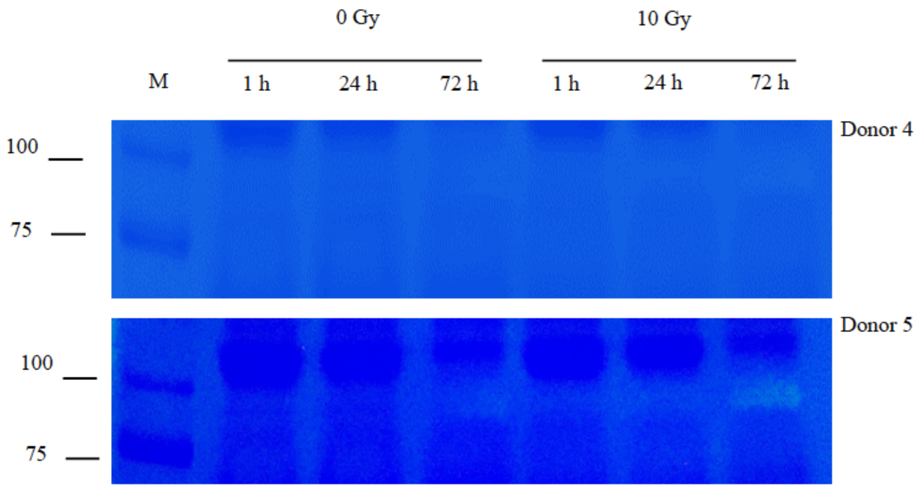

B

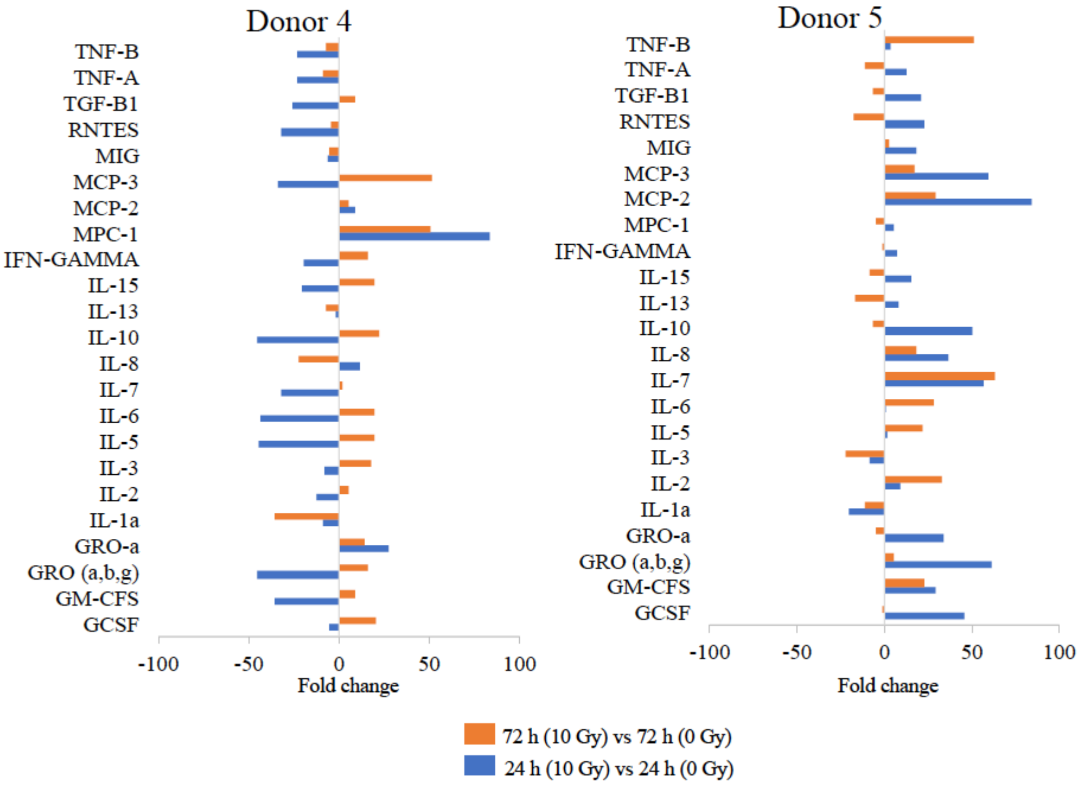

FIGURE 2S | Matrix Metalloproteases (MMP-9) and cytokine analysis for two selected donors. (A) The gelatinase activity of MMP-9 is demonstrated by the white band observed only in Donor 5 at 72 h, when PBMC are co-cultured with Caco-2 exposed to 10 Gy. (B) Cytokines secretion was evaluated as fold changes (FC) between the 10 Gy-irradiated and sham condition at the two different time-points, 24 h (blue) or 72 h (orange).

REFERENCES

1. Maccario C, Savio M, Ferraro D, Bianchi L, Pizzala R, Pretali L, Forti L, Stivala LA. The resveratrol analog 4,4′-dihydroxy- trans -stilbene suppresses transformation in normal mouse fibroblasts and inhibits proliferation and invasion of human breast cancer cells. *Carcinogenesis* (2012) 33(11):2172–2180 doi.org/10.1093/carcin/bgs244.

2. Pujada A, Walter L, Patel A, *et al.* Matrix metalloproteinase MMP9 maintains epithelial barrier function and preserves mucosal lining in colitis associated cancer. *Oncotarget* (2017) 8(55):94650–94665.

3. Strissel KJ, Rinehart WB, Fini ME. Regulation of paracrine cytokine balance controlling collagenase synthesis by corneal cells. *Invest Ophthalmol Vis Sci* (1997) 38(2):546-52.

4. Commins SP, Borish L, Steinke JW. Immunologic messenger molecules: cytokines, interferons, and chemokines. *J Allergy Clin Immunol.* (2010) 125(Suppl2):S53-72.

5. Ma Z, Chang MJ, Shah RC, Benveniste EN. Interferon-γ-activated STAT-1α suppresses MMP-9 gene transcription by sequestration of the coactivators CBP/p300. *J Leukoc Biol* (2005) 78:515–523.

6. Van den Steen PE, Proost P, Wuyts A, Van Damme J, Opdenakker G. Neutrophil gelatinase B potentiates interleukin-8 tenfold by aminoterminal processing, whereas it degrades CTAP-III, PF-4, and GRO-alfa and leaves RANTES and MCP-2 intact. *Blood* (2000) 96:2673-2681.
